# An evaluation of WHO emergency guidelines for Zika virus disease

**DOI:** 10.1111/jebm.12347

**Published:** 2019-06-18

**Authors:** Susan L Norris, Henry Louis, Veronica I Sawin, Teegwendé V Porgo, Yuk Hei Agnes Lau, Qi Wang, Mauricio Ferri

**Affiliations:** ^1^ WHO Guidelines Review Committee Secretariat World Health Organization Geneva Switzerland; ^2^ Department of Social and Preventative Medicine, Centre de Recherche du CHU de Québec—Hôpital de l'Enfant‐Jésus Laval University Quebec City Canada; ^3^ Department of Pharmacy University of California San Francisco California; ^4^ Evidence‐Based Medicine Center of Lanzhou University, Chinese GRADE Center—Lanzhou University Lanzhou People's Republic of China

**Keywords:** AGREE II, evaluation studies, evidence‐based practice, global health, guidelines, methods, quality control, World Health Organization, Zika virus disease

## Abstract

**Background:**

In the face of an unclear causal association between Zika virus *in utero* exposure and congenital abnormalities and urgent demand for guidance, the World Health Organization (WHO) had to produce timely and trustworthy guidelines during the 2016 Public Health Emergency of International Concern (PHEIC).

**Methods:**

This is a cross‐sectional evaluation of WHO emergency guidelines produced during the Zika virus disease PHEIC from 1 February to 18 November 2016. We assessed adherence to WHO publication requirements and the reporting of guideline development processes associated with trustworthiness. In the absence of quality appraisal tools for guidelines developed under compressed timeframes, we applied the Appraisal of Guidelines for Research and Evaluation (AGREE II) tool.

**Results:**

We included 21 guidelines (13 de novo and 8 updates). Six guidelines used a formal evidence review process. Most guidelines involved external experts in the development process and collected declarations of interest. Peer review was reported in six documents. Most emergency guidelines included updating plans. The highest scoring AGREE II domain was clarity of presentation (median score 78%); the lowest scoring domain was applicability (median score 18%).

**Conclusion:**

WHO developed moderate‐ to high‐quality emergency guidelines in the challenging context of a PHEIC. We found improvement opportunities for WHO guideline development teams in the use of evidence to formulate recommendations, the collection of declarations of interest, reporting of conflicts of interest, and the use of existing WHO organizational quality assurance processes.

## 1 INTRODUCTION

Zika virus (ZIKV) disease and its multiple long‐term complications (eg, congenital malformations and developmental abnormalities) continue to impose a considerable burden on affected communities, constituting an enduring challenge for public health authorities at global and local levels. As of February 2018, 86 countries had evidence of ongoing or past vector‐borne ZIKV transmission^1^ and 31 countries had reported cases of microcephaly and other central nervous system malformations (March 2017).^2^ The incidence varies greatly across the globe as ZIKV is primarily transmitted by the bite of infected Ades mosquitoes.^3^ For example, the incidence rates (per 100 000 population) reported in the Americas from 2015 to 2018 for ZIKV disease and the number of live newborns who met the criteria for suspected congenital syndrome associated with ZIKV infection were 0.06 and 103 in North America, 176.1 and 2952 in Brazil, and 411.0 and 27 in non‐Latin Caribbean countries.^4^

There is no treatment available for ZIKV infection,^3^ thus the current state‐of‐the science for ZIKV disease management is centered on public health interventions to limit disease transmission to vulnerable populations and to provide supportive care for newborns and infants with congenital malformations and developmental abnormalities^5^, ^6^, ^7^, ^8^ requiring prolonged allocation of finite health care resources.^9^, ^10^ Furthermore, the implications for blood product safety and maternal, child and reproductive health highlight the cross‐cutting nature of ZIKV disease and yet again reveal deficiencies in international preparedness and capacity to respond to biologic threats.

In the early phases of the 2015‐2016 ZIKV disease outbreak in the Americas, the causal association between *in utero* exposure to ZIKV and congenital abnormalities was unclear^7^ leaving public health authorities without a clear path to produce timely, high‐quality and trustworthy guidelines. Urgent demand, scarcity of structured scientific data, political instability in affected areas leading to uncertainty about implementation capability, and the need to produce multiple guidelines concurrently were additional challenges besetting WHO ZIKV disease guidelines apart from traditional clinical or public health guidelines. Finally, there is no international consensus on the optimal processes and methods for developing such guidelines. In response to these challenges, the WHO Guidelines Review Committee Secretariat and the Department of Pandemic and Epidemic Diseases launched emergency guideline templates and prototypical guideline development processes which built on lessons learned with other recent public health emergencies.^11^

The objective of this study was to describe the characteristics related to trustworthiness of WHO ZIKV guidelines published in response to the ZIKV Public Health Emergency of International Concern (PHEIC) in 2016, and to assess their quality in terms of stakeholder involvement, rigor of development, clarity of presentation, inclusion of implementation considerations, and management of conflicts of interests.

## 2 METHODS

We examined WHO guidelines developed and published by WHO headquarters specifically for the ZIKV outbreak response in 2016. We defined the study period, 1 February to 18 November 2016, based on WHO's declaration that the association of ZIKV infection with clusters of microcephaly and other neurological disorders constituted a PHEIC on 1 February 2016, and the end of the PHEIC on 18 November 2016.^10^ According to WHO's definition of a guideline,^12^ documents were so classified if they contained original health recommendations irrespective of label or development processes. We excluded other types of documents such as action plans, media releases, fact sheets, situation reports and guidelines not aimed at the ZIKV outbreak response. We electronically searched WHO's global digital library (Institutional Repository for Information Sharing [IRIS]) and e‐Pub (WHO's internal tracking and approval system for all information products) using the text word “Zika”, hand‐searched WHO websites and consulted key WHO staff in relevant technical units. Two independent reviewers assessed and classified each document retrieved as to whether it fulfilled the WHO definition of a guideline and other study selection criteria.

We extracted guideline characteristics and information on publication format. In addition, we tracked updates throughout the study period, identified translations to regionally important languages, and described guideline quality assurance and control efforts such as external peer review, Guidelines Review Committee (GRC) approval (WHO's internal quality control process for guidelines) or presence in e‐Pub.

We also identified and extracted data on the characteristics, format and related text for discrete recommendations within each included guideline. WHO defines recommendations as statements that tell end‐users what to do in specific situations to achieve the best health outcomes.^12^ We further defined discrete recommendations as easily identifiable statements that were distinct or separated from the body of the guideline text or clearly labeled as recommendations.

All data were extracted by one coauthor and independently checked by a second one. This study did not undergo ethics review.

### 2.1 Guideline quality assessment

Although validated quality assessment tools specific for emergency guidelines are not available in the medical literature,^13^ the same principles for quality apply to all types of guidelines. Therefore, for this evaluation, we assumed that the guideline trustworthiness principles of transparency; minimization of risk of bias in the assessment of primary studies, synthesis of evidence and in the formulation of recommendations; and the presentation of implementable recommendations apply to all types of guidelines and recommendations (Table 1).^14^

**Table 1:** Essential steps for developing trustworthy guidelines

1.	Guideline development processes and funding sources need to be detailed and accessible.
2.	Contributors need to disclose all relevant interests, and conflicts need to be appropriately managed.
3.	The guideline development group should be multidisciplinary and balanced, including relevant stakeholders and persons affected by the recommendations.
4.	High‐quality evidence reviews should underpin recommendations.
5.	Each recommendation should be accompanied by a rationale statement, an assessment of the certainty of the evidence, the strength of the recommendation, and any differences in opinion among the guideline development group members.
6.	Recommendations should be clearly articulated and precise.
7.	External review of the draft guideline should encompass a full spectrum of relevant stakeholders.
8.	Plans for updating should be included and emerging data should be monitored.

*Note*: Based on Ref. (14).

In addition, four independent appraisals of each guideline were performed using the Appraisal of Guidelines for Research and Evaluation (AGREE II) instrument.^15^, ^16^ This widely accepted tool contains 23 items across six domains: scope and purpose; stakeholder involvement; rigor of development; clarity of presentation; applicability; and editorial independence. The appraisers were trained to use AGREE II in accordance with the latest version of the instrument manual.^17^

We report the prevalence of key characteristics and present AGREE II scores for each of the six domains for each guideline (scaled to a percentage of the maximum score) and then report scores across guidelines using the median value and the interquartile range for each domain. All statistical analyses were conducted using Stata 12.1 (StataCorp LP, College Station, Texas, USA).

## 3 RESULTS

**Figure 1:**
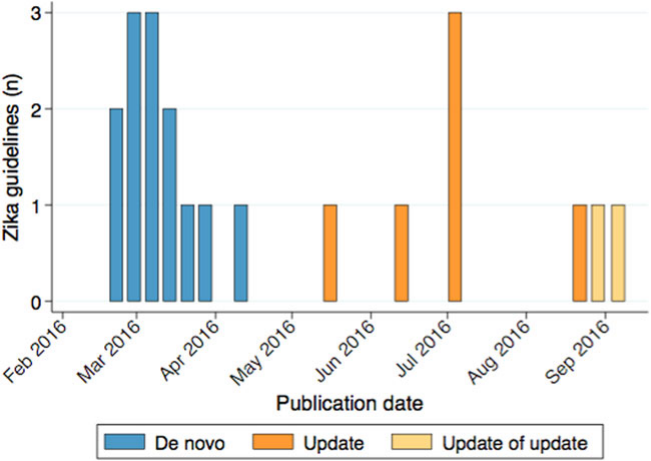
Zika virus disease guidelines publication timeline *Note*: The Public Health Emergency of International Concern was declared on 1 February 2016.

We identified 21 WHO ZIKV guidelines published during the study period, of which 13 were developed for the first time (de novo) and 8 were updates (Table 2). De novo guidelines were published an average of 34 (SD 14) days after the declaration of the PHEIC on 1 February 2016, while updates were published an average of 164 (SD 42) days after this declaration (Figure 1). Fourteen (66%) guidelines had both Portuguese and Spanish translations and 13 (62%) had French. All guidelines were available in downloadable format from WHO's ZIKV webpage. Of WHO's publication requirements, 19 guidelines (90%) met all six requirements, while two guidelines did not use WHO's emergency guideline template and one of these two guidelines also did not contain the required reference number (Table 2).

**Table 2:** Zika virus disease guidelines: characteristics related to publication type and format

Characteristic	Total n = 21
De novo guideline, n (%)	13 (62)
Updated guideline, n (%)	8 (38)
Days since PHEIC declared to publication, mean (SD)	
All guidelines	78 (69)
De novo guidelines	34 (14)
Updates	164 (42)
Days between de novo and update publication, mean (SD)	110 (37)
Translations, n (%)	
Portuguese	14 (66)
Spanish	14 (66)
French	13 (62)
Publication format, n (%)	
Used the WHO emergency guideline template	19 (90)
Downloadable version available	21(100)
Included a WHO reference number	20 (95)
Included the WHO logo	21(100)
Included the WHO legal disclaimer	21 (100)
Included the publication date	21 (100)
Number of pages, mean (range)	9 (2‐42)

*Note*: n, number; PHEIC, Public Health Emergency of International Concern; WHO, World Health Organization. A list of the 21 guidelines is found in Annex 1.

In terms of development processes and characteristics related to quality, approximately half of the guidelines reported performing an evidence review; however, details of the evidence review methods (eg, systematic review, rapid review, nonsystematic search) were rarely described. All six guidelines (29%) that reported using a systematic or rapid review were updates; none of the de novo guidelines mentioned a systematic or rapid review. The majority (57%) of the guidelines referenced other WHO guidelines and all cited at least one other document or publication (Table 3).

**Table 3:** Zika virus disease guidelines: characteristics related to quality

Characteristic	Total n = 21, n (%)
Reported scope	
Target audience indicated	20 (95)
Setting(s) indicated	12 (57)
Used evidence	
Contained at least one reference document	21 (100)
Referenced any type of review	10 (48)
Included a systematic or rapid review	6 (29)
Referenced other WHO guidelines	12 (57)
Used GRADE	1 (5)
Used structured approach for formulating recommendations	4 (30)
Involved external experts in any function	18 (86)
Involved external experts to develop recommendations	13 (62)
Collected declarations of interest	17 (81)
Managed conflicts of interest	17 (81)
Disclosed funder(s)	4 (20)
Indicated expiration date or planned update	17 (81)
Subject to quality control	
Approved by the WHO GRC	1 (5)
Included in e‐Pub	16 (76)
Peer reviewed	6 (29)

*Note*: GRADE, Grading of Recommendations Assessment, Development and Evaluation; GRC, Guidelines Review Committee; n, number of guidelines; WHO, World Health Organization. A list of the 21 guidelines is found in Annex 1.

Most guidelines involved external experts in many functions; 62% of guidelines included external experts as members of guideline development group (ie, responsible for formulating recommendations) with their names listed in the document. Of the 21 guidelines reviewed, 17 (81%) reported collecting declarations of interest (DOI). Of the four guidelines that did not report collecting DOI, three did not include external experts in the formulation of recommendations, involving them rather as peer reviewers. Four guidelines (20%) reported funding sources. External peer review was reported in six (29%) guidelines and 81% included an expiration date or plan for updating. One guideline (5%) was reviewed and approved by the WHO Guidelines Review Committee.

**Figure 2:**
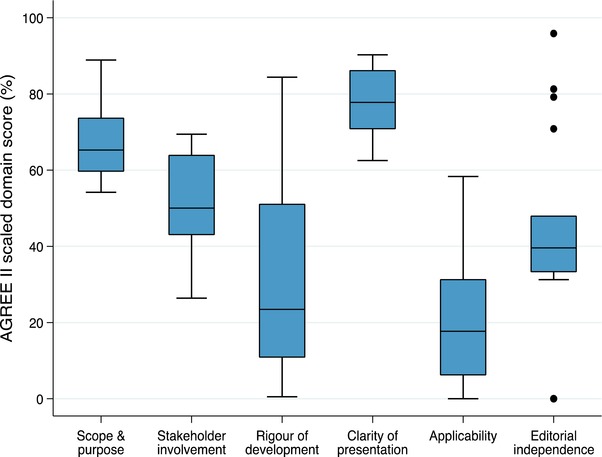
Zika virus disease guidelines AGREE II scores *Note*: AGREE II quality domain scores (vertical axis) are based on standardized scores of the four independent reviewers for each domain (horizontal axis) (11‐13). The boxes represent the interquartile ranges, and the middle line the median score. The whiskers represent the ranges.

The highest scoring AGREE II domains were clarity of presentation (median score 78% (interquartile range [IQR] 71%‐86%). The lowest scoring domain was applicability (18% [IQR 6%‐31%]). There was substantial variability in most of the domains, most notably for rigor of development (Figure 2).

For the recommendation‐level assessment, we identified 58 discrete recommendations contained in 6 of the 13 guidelines that were current at the time of data extraction (November 2016) (Table 4). The remaining then‐current guidelines did not have recommendations that could be identified as discrete or stand‐alone statements distinct from the body of the text. Fifty of these 58 recommendations (86%) came from guidelines that used systematic reviews or some other type of evidence review. Most recommendations identified the target population, 31 described the recommended intervention in detail, and none provided a comparator to the recommended intervention. About half of the recommendations explicitly considered decision‐making criteria in addition to benefits and harms during the formulation phase. Half of the recommendations were contained in a guideline that included an implementation tool.

**Table 4:** Zika virus disease guidelines: recommendation‐level characteristics

Characteristic	n (%)
Recommendation extracted from a guideline that used some type of evidence review	50 (86)
Recommendation extracted from a guideline that used a systematic review	40 (69)
Elements of the recommendation described or identified^a^	
Population	56 (96)
Intervention components	31 (53)
Comparator	0 (0)
Rationale linking evidence to recommendation	8 (14)
Conditions and applicability	20 (34)
Evidence‐to‐decision considerations reported^a^	
Values and preferences	33 (57)
Harms, unintended consequences	30 (52)
Costs	1 (2)
Impact on equity	35 (60)
Feasibility	30 (52)
Implementation tools available	29 (50)
Dissemination plan included or linked	1 (2)

*Note*: Discrete recommendations were identified and extracted from 13 of 21 WHO ZIKV guidelines. n, number of recommendations; total n = 58 recommendations from 6 of the 13 guidelines that were current at the time of data extraction (November 2016). The six guidelines are identified in Annex 1.

a Assessment by one reviewer.

## 4 DISCUSSION

Our study suggests that WHO can efficiently and effectively develop and publish timely, moderate‐ to high‐quality guidelines in the very challenging context of a biologic threat leading to a PHEIC. Most of the guidelines produced by WHO in response to the 2016 ZIKV disease outbreak used the organizational template and complied with publication and reporting requirements. These guidelines performed particularly well on key aspects of trustworthiness such as reporting of DOI and management of conflicts of interest and scored high in the AGREE II quality domains of “scope and purpose” and “clarity of presentation”. Nonetheless, we identified opportunities for improvement in many components of the guideline development process pertaining to trustworthiness: the use of evidence such as from systematic reviews, collection DOI and transparent management of conflicts of interest of all contributors, reporting the role of funders, more inclusive and explicit consideration of decision elements beyond benefits and harms (ie, using comprehensive evidence‐to‐decision tables), consistent peer review of draft final guidelines, the development of tools for implementation in the local context and the uniform use of quality assurance processes. WHO guidelines produced in the context of the ZIKV disease outbreak were rarely submitted to the GRC or its Secretariat: some form of quality assurance is essential for all of WHO's information products, regardless of the context, whether this is the GRC or some other closely related process. In addition, all WHO information products should be registered in the publication clearance and approval system. These are thus important areas for improvement which WHO is currently addressing.

Significant challenges when evaluating emergency guidelines include the poor reporting of development methods in these documents and the lack of validated and widely accepted standards and quality assessment tools.^13^ Our evaluation suggests considerable improvement of the reporting of ZIKV guidelines when compared to WHO emergency guidelines produced for other recent outbreak‐related PHEICs.^11^ Although we cannot determine the causes of this improvement, the accumulating experience of the technical units in developing emergency guidelines and the implementation of an emergency guideline template and development process early in the response may explain in part these findings.

Standards for the assessment of guideline quality such as the components for trustworthy guidelines^14^ and AGREE II,^15^, ^16^, ^17^ were developed for clinical guidelines in the nonemergency context where there is a longer time‐period available for development, knowledge gaps and end‐user needs may be much clearer, there may be more (high‐quality) structured scientific evidence, and the implementation settings and health systems will likely be less fragile and chaotic. Thus, the applicability of these tools to emergency guidelines may be limited and our evaluations must be interpreted with caution. Certainly, these tools should not be used to determine if a specific emergency guideline is trustworthy, high‐quality and impactful in areas affected by ZIKV disease outbreaks.

The high scores in the AGREE II quality domains of “scope and purpose” and “clarity of presentation” are similar to those reported in a previous evaluation of WHO emergency guidelines.^11^ These high scores may be attributable to the operational focus of both groups of guidelines, requiring narrow and specific health questions, clear target populations and a concise format. In addition, the items that constitute these two AGREE II domains were clearly described in the template used for most of the guidelines in our study. Our findings are also consistent with a previous evaluation of standard WHO guidelines produced outside of emergency situations,^18^ thus the high scores in these domains may reflect other aspects of WHO guideline processes that were not captured in our study.

The results of the AGREE II quality domain of “applicability” showed significant room for improvement, however. This domain covers the barriers and facilitators to implementation, strategies to improve uptake, cost and implementation tools.^17^ The absence of these items in the guideline template used during this outbreak, as well as the scarce knowledge about ZIKV disease and the extreme uncertainty in the field may have contributed to this finding. Failing to include these criteria in emergency guidelines may represent a major shortcoming with important pragmatic and operational consequences.

WHO ZIKV disease emergency guidelines also had suboptimal performance on the AGREE II domain of “rigor of development.” This finding is likely due to the infrequent use and availability of scientific evidence, and the failure to use a structured decision‐making process for formulating recommendations. These components of guidelines are corner‐stones of trustworthiness, and actions to improve them need to be prioritized in future iterations of the templates and development processes.

We did not evaluate many important questions such as how to prioritize topics for guideline development during an emergency, how to assess existing guidelines for relevance and applicability, optimal dissemination methods and tools, the uptake by end‐users, and most importantly, the impact on health outcomes of the affected populations. We also did not address the validity of the recommendations issued in the emergency context. As new data and updated guidelines become available, the validity of the previous recommendations and the robustness of the processes used to formulate them should be assessed and the results used to improve processes and methods in future.

WHO has an intentionally broad definition for guidelines, which may have resulted in misclassification of some information products as guidelines when the purpose of the document was not to provide normative guidance. However, we had a clear operational definition and eligibility criteria for guidelines and 90% of the included documents used the emergency interim guidance template, suggesting that misclassification was likely rare. Another potential limitation was our reliance on information that was reported in guidelines; we did not query guideline authors for additional information. We chose to focus primarily on rigor of quality of development as well as WHO's reporting requirements; we did not apply the CheckUp checklist for reporting of updated guidelines although many of its items were encompassed by our evaluation.^19^

## 5 CONCLUSION

WHO developed moderate‐ to high‐quality emergency guidelines in the challenging context of a PHEIC. We found improvement opportunities for WHO guideline development teams in the use of evidence to formulate recommendations, the collection of DOI, reporting and management of conflict of interest, and the use of existing WHO organizational quality assurance processes. It is important to ensure that the lessons learned in this evaluation are translated into WHO's standard operational procedures and made available to technical units responsible for developing emergency guidelines in the future. Equally important is the establishment and continuous refinement of emergency guideline development methods and accompanying tools to facilitate production of the best possible guidance in a timely and trustworthy manner.

## COMPETING INTERESTS

SLN is a member of the Grading of Recommendations Assessment, Development and Evaluation (GRADE) Working Group and has published numerous papers related to GRADE. GRADE is the guideline process used by her employer, the World Health Organization, to develop guidelines. MF is currently an employee of Bristol Myers Squibb and owns company stock as part of his remuneration plan; a family member was employed by Bristol Myers Squibb at the time this study was performed. YHAL is currently an employee at Genentech and owns company stock as part of her renumeration; she had no affiliation with Genentech at the time the study was performed. The other authors declare no competing interests.

## DISCLAIMER

The authors alone are responsible for the views expressed in this article and they do not necessarily represent the views, decisions, or policies of the institutions with which they are affiliated.

## Supporting information

Online Annexure

Supporting Material

## References

[1] WHO . Zika Virus Classification Table, Data as of 15 February 2018. http://apps.who.int/iris/bitstream/handle/10665/260419/zika-classification-15Feb18-eng.pdf;jsessionid=4D68513997BB454F75826D2BABF4F73F?sequence=1 (accessed 10 October 2018).

[2] WHO . Zika virus , Microcephaly and Guillain‐Barre Syndrome situation report. Geneva: World Health Organization; 2017 (http://apps.who.int/iris/bitstream/10665/254714/1/zikasitrep10Mar17-eng.pdf?ua=1 (accessed 10 October 2018).

[3] WHO . Zika Virus: Key facts 20 July 2018. https://www.who.int/en/news-room/fact-sheets/detail/zika-virus (accessed 27 December 2018).

[4] Pan American Organiztion and the World Health Organization . Zika cases and congenital syndrome associated with Zika virus reported by countries and territories in the Americas, 2015 ‐ 2018. Cumulative cases. https://www.paho.org/hq/index.php?option=com_docman&view=download&category_slug=cumulative-cases-pdf-8865&alias=43296-zika-cumulative-cases-4-january-2018-296&Itemid=270&lang=en (accessed 27 December 2018).

[5] Petersen LR , Jamieson DJ , Powers AM , Honein MA . Zika virus. N Engl J Med. 2016; 374:1552–1563. 27028561 10.1056/NEJMra1602113

[6] De Oliveira WK , Carmo EH , Henriques CM , et al. Zika virus infection and associated neurologic disorders in Brazil. N Engl J Med. 2017;376:1591–193. 28402236 10.1056/NEJMc1608612 PMC5544116

[7] Broutet N , Krauer F , Riesen M , Khalakdina A , Almiron M . Zika virus as a cause of neurologic disorders. N Engl J Med. 2016;374:1506–1509. 26959308 10.1056/NEJMp1602708

[8] Brito CA , Brito CC , Oliveira AC , et al. Zika in Pernambuco: rewriting the first outbreak. Rev Soc Bras Med Trop. 2016;49:553–558. 27812648 10.1590/0037-8682-0245-2016

[9] Lee BY , Alfaro‐Murillo JA , Parpia AS , et al. The potential economic burden of Zika in the continental United States. PLoS Negl Trop Dis. 2017 11(4): e0005531 10.1371/journal.pntd.0005531. 28448488 PMC5407573

[10] World Health Organization . WHO Zika Strategic Response Plan, Revised for July 2016‐December. Geneva, Switzerland: WHO Press; 2016.

[11] Norris SL , Ivey Sawin V , Ferri M , Reques Sastre L , Porgo TV . An evaluation of emergency guidelines by the World Health Organization in response to four infectious disease outbreaks. PLoS ONE. 2018; 13(5). 10.1371/journal.pone.0198125. PMC5976182 29847593

[12] World Health Organization . WHO Handbook for Guideline Development (2nd edition). Geneva, Switzerland: WHO Press; 2014.

[13] Siering U , Eikermann M , Hausner E , Hoffmann‐Eßer W , Neugebauer EA . Appraisal tools for clinical practice guidelines: a systematic review. PLoS ONE. 2013 8(12): e82915 10.1371/journal.pone.0082915. 24349397 PMC3857289

[14] Institutes of Medicine . Clinical Practice Guidelines We Can Trust. Washington (DC): National Academies Press (US); 2011. doi: 10.17226/13058.

[15] Brouwers MC , Kho ME , Browman GP , et al. on behalf of the AGREE Next Steps Consortium . Performance, usefulness and areas for improvement: development steps towards the AGREE II—Part 1. Can Med Assoc J. 2010;182:1045–1052. 20513780 10.1503/cmaj.091714 PMC2900328

[16] Brouwers MC , Kho ME , Browman GP , et al. on behalf of the AGREE Next Steps Consortium . Validity assessment of items and tools to support application: development steps towards the AGREE II—Part 2. Can Med Assoc J. 2010;182:E472–E478. 20513779 10.1503/cmaj.091716 PMC2900368

[17] Brouwers MC , Kho ME , Browman GP , et al. on behalf of the AGREE Next Steps Consortium. AGREE II: Advancing guideline development, reporting and evaluation in healthcare. Can Med Assoc J. 2010; 182:E839–E842. 20603348 10.1503/cmaj.090449 PMC3001530

[18] Burda BU , Chambers AR , Johnson JC . Appraisal of guidelines developed by the World Health Organization. Public Health. 2014;128(5):444–474. 24856197 10.1016/j.puhe.2014.01.002

[19] Vernooij RWM , Alonso‐Coello P , Brouwers M , Martínez García L , CheckUp Panel . Reporting items for updated clinical guidelines: checklist for the reporting of updated guidelines (checkup). PLoS Med 2017; 14(1): e1002207 10.1371/journal.pmed.1002207. 28072838 PMC5224740

